# Behavioral and Physiological Evidence of a favored Hand Posture in the Body Representation for Action

**DOI:** 10.1093/cercor/bhab011

**Published:** 2021-02-22

**Authors:** Daniele Romano, Alessandro Mioli, Marco D’Alonzo, Angelo Maravita, Vincenzo Di Lazzaro, Giovanni Di Pino

**Affiliations:** Psychology Department, NeuroMi, Milan Center for Neuroscience, University of Milano-Bicocca, Milan, Italy; Research Unit of Neurophysiology and Neuroengineering of Human-Technology Interaction (NeXTlab), Department of Medicine, Università Campus Bio-Medico di Roma, Rome, Italy; Research Unit of Neurophysiology and Neuroengineering of Human-Technology Interaction (NeXTlab), Department of Medicine, Università Campus Bio-Medico di Roma, Rome, Italy; Psychology Department, NeuroMi, Milan Center for Neuroscience, University of Milano-Bicocca, Milan, Italy; Research Unit of Neurology, Neurophysiology and Neurobiology, Department of Medicine, Università Campus Bio-Medico di Roma, Rome, Italy; Research Unit of Neurophysiology and Neuroengineering of Human-Technology Interaction (NeXTlab), Department of Medicine, Università Campus Bio-Medico di Roma, Rome, Italy

**Keywords:** body representation, body schema, hand posture, motor control, TMS

## Abstract

Motor planning and execution require a representational map of our body. Since the body can assume different postures, it is not known how it is represented in this map. Moreover, is the generation of the motor command favored by some body configurations? We investigated the existence of a centrally favored posture of the hand for action, in search of physiological and behavioral advantages due to central motor processing. We tested two opposite hand pinch grips, equally difficult and commonly used: forearm pronated, thumb-down, index-up pinch against the same grip performed with thumb-up. The former revealed faster movement onset, sign of faster neural computation, and faster target reaching. It induced increased corticospinal excitability, independently on pre-stimulus tonic muscle contraction. Remarkably, motor excitability also increased when thumb-down pinch was only observed, imagined, or prepared, actually keeping the hand at rest. Motor advantages were independent of any concurrent modulation due to somatosensory input, as shown by testing afferent inhibition. Results provide strong behavioral and physiological evidence for a preferred hand posture favoring brain motor control, independently by somatosensory processing. This suggests the existence of a baseline postural representation that may serve as an a priori spatial reference for body–space interaction.

## Introduction

We all use our hands daily for a multitude of scopes, from object manipulation to interpersonal contacts, thus experiencing every possible posture. During hand actions, we are extremely quick and precise in choosing and shaping grips and assuming postures (think, for example, pantomiming), even without full attentional or sensory (e.g., visual) control.

We are also able to localize precisely sensory stimuli delivered to the hand in the space and couple them with spatial visual information. Such abilities suggest that our brain owns a precise, dynamic spatial representation of our body, the so-called body representation. Body representation is critical to identify the bodily self and helps guide body interactions with the external world (De Vignemont 2010), suggesting that spatial information should be deeply embedded in the body representation.

Motor control is modeled through algorithms implementing a forward model that makes a prediction of action outcome, an inverse model which estimates the motor command required to achieve the desired outcome and an internal model, where kinematic and dynamic features of the body and its interactions with the environment are stored (Kawato 1999; Wolpert and Ghahramani 2000).

By both tapping into the cognitive literature of the body schema and into the motor control modeling literature, planning and executing a movement seem to require a representational map of our body. In this map, the body can be configured in several ways, accordingly with the different postures which it can actually assume. Moreover, the body posture is by itself a key parameter that has to be estimated and controlled during any action.

When the central nervous system plans to execute a movement and recall the map of the body, it is not known whether all postures are equivalent or if there are some body configurations that favor the generation of the motor command.

This study was designed to investigate the existence of a centrally favored posture of the hand for action, independently from any advantage due to biomechanics, sensory feedback, and peripheral and/or subcortical processes. To focus the investigation on the hand, we sought for the prototypical orientation that the hand would have while grasping. Being thumb and the index finger crucial for both power and precision grips (Napier 1962; MacKenzie and Iberall 1994), we decided to compare two equally frequently used postures: the pinch grip performed in the thumb-down posture with the opposite thumb-up pinch.

To the above outcomes, we tested at multiple levels the facilitation of the motor system to perform actions, with four experiments in healthy people.

In “Experiment 1,” we implemented a behavioral task where participants were asked to grasp as fast as possible a cube in front of them, once a visual cue showed the pinch configuration to use (i.e., thumb-up/finger-down or vice versa). Reaction time (RT) for movement onset measured movement computation speed, while the time needed from the onset to reach the cube was taken as a cue of functional advantage of each posture.

In “Experiment 2,” corticospinal excitability was tested through transcranial magnetic stimulation (TMS) of the primary motor cortex (M1) by recording the evoked muscle responses (motor evoked potentials [MEPs]) from two muscles: one mainly involved in holding the pinch posture (i.e., the first dorsal interosseous [FDI]) and the other in shaping the posture (i.e., the abductor pollicis brevis [APB]).

With this experiment, we aim to infer M1 cortex area excitability in the two different postures. However, performing different postures may result in different levels of pre-stimulus muscle contraction, which, together with the TMS-induced corticospinal descending activity, is known to affect MEP amplitude (Di Lazzaro et al. 2000). To control for this potential confounding factor, we compared changes of MEP amplitude respectively with changes of the pre-stimulus ongoing muscular activity computed as the root mean square (RMS) of the 1.8-s pre-stimulus electromyographic (EMG) activity in the two postures.

Besides being independent of the pre-stimulus muscular activity, any grip posture advantage based on motor programming would also be independent of the actual motor output. In order to test the potential facilitation of motor system before any movement is acted, in “Experiment 3,” corticospinal excitability was measured with the hand at rest in a neutral position, while participants performed three different mental representations of action tasks: 1) observation; 2) imagination, and 3) preparation of the pinch grip in the thumb-down or thumb-up configuration.

Furthermore, we recently found consistent hand posture-related advantages in sensory processing for the thumb-up posture (Romano et al. 2017).

A facilitation of the motor system for a given posture would suggest the following alternatives: 1) the sensory facilitation for tactile processing and motor postural advantages are entangled in a way that sensory stimulation affects motor facilitation, suggesting for a sensory–motor integration mechanism underlying the effect; 2) conversely, facilitations of sensory and motor systems are independent, so that the cause of facilitation resides at a higher level than single sensory and motor processing. In the latter case, a high-level multimodal representation of body posture may likely impact on the two functions independently.

We tested those two alternative hypotheses in “Experiment 4,” where we employed the short-latency afferent inhibition (SAI), a sensory–motor integration protocol testing the cholinergic inhibition of cortical motor output induced by the somatosensory afferences (Di Lazzaro et al. 2000). In the SAI, an electrical conditioning stimulus of the median nerve at the wrist preceded the M1 TMS pulse by an interval locked with the latency of the cortical somatosensory potential (N20). This interval represents the time needed by the afferent inputs to reach the somatosensory cortex. SAI measures the percentage of MEP inhibition when TMS is preceded by the afferent preconditioning stimulus. Thus, different SAI inhibition in one of the two postures would mean that any motor facilitation we could find would be just the effect of a different sensory processing; sensory facilitation would induce a higher motor inhibition. This would be in favor of the first alternative, while the absence of a SAI difference between postures would support the latter alternative.

## Materials and Methods

### Participants

Forty participants were enrolled for the Experiment 1 and randomly divided into two different groups depending on the starting position of the right hand used to complete the task: prone for 20 participants (10 male; 10 female; mean age: 25.45; standard deviation [SD]: 2.95) and supinated for the other 20 (10 male; 10 female; mean age: 29.90; SD: 5.08). Sixteen participants participated in Experiments 2*–*4 of the study (7 female; mean age: 27.1 years, range: 21–36). The same participants were involved in all the three Experiments 2–4. The study was approved by the local ethics committee (EMBODY protocol). All participants were right-handed (self-report), and they expressed their informed consent to the experiment. The entire experimental session of Experiments 2– 4 took around 3 h including the breaks between the three consecutive tasks. Data from Experiment 4 (SAI) of two participants were excluded from the analysis due to excessive EMG artifacts, so that the final sample of Experiment 4 is 14. The datasets generated during this study are available at Mendeley Data (Dataset_Romano_CerebralCortex_2020).

### Experiment 1

**Figure 1:**
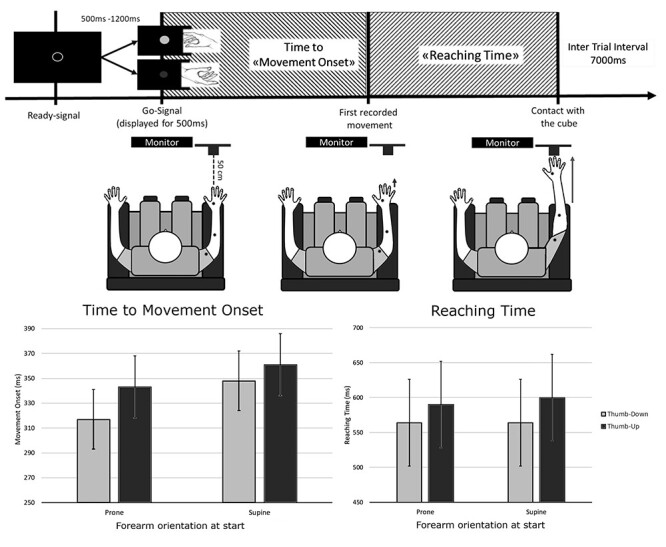
Experiment 1 setup and results. The upper panels schematically show the Experiment 1 setup and procedure. Movement onset: The time from the go-signal to the very first movement recorded by the three sensors (black dots). Reaching time: The time that separates the movement onset from the first contact with the target. The lower panels show Experiment 1 results split according to the initial orientation of the arm. The time recorded by the sensors is averaged because the factor Sensor did not interact with the forearm orientation and the hand posture. Error lines indicate the 95% CIs. The thumb-down pinch starts earlier than thumb-up both when the forearm starting position was prone and supine (left graph: movement onset). The thumb-down pinch had faster reaching time independently from the forearm starting orientation and the movement onset (right graph: reaching time).

Three magneto-inertial sensors (Delsys Trigno, Natick, MA) were placed on the participant’s right arm: one on the dorsum of the hand at the level of the FDI, one on the forearm at the level of the flexor digitorum superficialis, and the last one at the level of the biceps. The participant sat on a chair, with their right arm on an armrest (Fig. 1). The experimental task consisted of 50 randomized grasping actions, 25 were done with a thumb-down posture and the remaining 25 with a thumb-up position. In each trial, participants had to grasp a target object (wooden cube, side: 4 cm) in front of them at 50 cm of distance, after a visual go-signal delivered through a PC monitor presented by a computerized software (OpenSesame). The go-signal consisted in a colored circle (diameter: 24 cm) on a black background presented at the center of the screen for 500 ms, preceded by a ready signal: an empty circle with white borders which randomly lasted from 500 to 1200 ms. The inter-trial interval was 7000 ms, during which the participant had to bring his arm back to the resting position. The color of the go-signal (yellow or blue) indicated the participant to grasp the target object with one or the other posture (thumb-down or thumb-up). The target object had a force-sensitive resistor (FSR) sensor mounted on both its upper and lower surfaces, to detect the exact time of touch by the participant. After having showed to the participants the two different postures, we gave them the instruction to pinch the cube with the posture instructed by the visual cue, as soon as they receive the cue. No emphasis was put to do fast or ballistic movements.

The initial movement of the body segments (i.e., hand, forearm, and arm) was detected using the modulus of the 3D accelerometer (sampling rate: 148 Hz). The threshold for movement detection was calculated as the mean measurement of the signal recorded 200 ms before the go-signal (resting phase) + 5^*^SD (Wentink et al. 2014).

As regards to the detection of touch on the target object, the FSR signal (sampling rate: 512 Hz) was filtered by a notch filter at 50 Hz, and a fixed threshold was employed to identify the time of touch. The selected threshold corresponded to 200 mN.

#### Analysis

After data visual inspection, trials were manually rejected when the 3D accelerometer clearly indicated a movement of the participant’s hand 200 ms before the go-signal (resting phase) (prone group [8.23%], supinated group [6.83%]). Trials were discarded also if the FSR sensor did not detect any touch by the participant (rejection rate was 0.95% for the pronated group and 1.5% for the supinated group).

Inferential statistics were performed through linear mixed models (LMMs) with the statistical software R (Team RC 2013) using the package lme4. We used the RT to initiate the action recorded by the accelerometers as dependent variable. Participants were added to the model as random effect variable. The model was analyzed with an analysis of variance (ANOVA) with Satterthwaite approximation for degrees of freedom as in Experiment 1. We entered, as fixed-effect model, a 2X (“Posture”: thumb-down/thumb-up) 3X (“Sensor position”: hand/forearm/arm) 2X (“forearm orientation”: prone/supine) full factorial design.

Additionally, we measured the reaching component of the action by calculating the delta between the onset of action and the first contact with the cube on a trial-by-trial basis (delta RT). This method gives an index of the behavioral advantage for a posture that is independent of the eventual difference of the initial computation of the action. We entered the delta RT as dependent variable of an independent LMM. Participants were modeled as random effect variable. The model was analyzed with an ANOVA with Satterthwaite approximation for degrees of freedom. The fixed-effect model included a 2X (Posture: Thumb-Down/Thumb-Up) 2X (forearm orientation: prone/supine) full factorial design.

### Experiment 2

**Figure 2:**
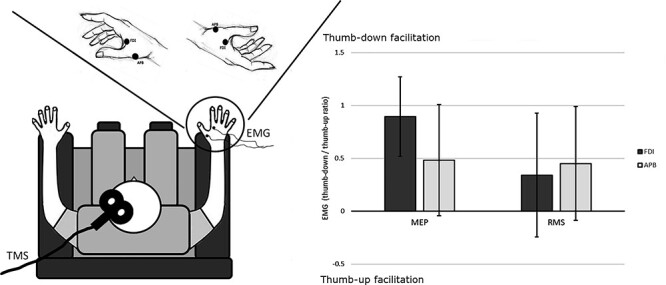
Experiment 2 setup and results. Left panel: Schematic representation of Experiment 2 setup. The hand postures (thumb-down or thumb-up) are depicted in the upper section. Each black dot shows the position of the pair of electrodes used for the FDI and APB EMG recording. Right panel: Experiment 2 results. Bars represent the average standardized EMG activity (EMG thumb-down/EMG thumb-up). Error lines indicate 95% CIs. MEP = EMG evoked by TMS; RMS = root mean square of the tonic basal EMG activity, computed 1.8 s before TMS stimulus. CIs above 0 indicate motor facilitation for the thumb-down posture (FDI-MEP). When the CIs cross the axis, there is no clear evidence for the facilitation of one of the two postures (APB-MEP and both RMS).

Participants were sitting on a comfortable chair. The elbow was resting on the armrest. MEPs were collected while the right hand was held in two different postures in independent blocks. In one block, the hand was held with the thumb in a lower position opposite to all the other fingers that were occupying an upper elevation modeling a c-shape configuration, as for preparing a pinch grip. This posture was found to induce faster and more accurate tactile discrimination (Romano et al. 2017). In the other block, the hand was held upside down, thus with the thumb above all the other fingers (Fig. 2). Participants were asked to maintain this position with the thumb-index pinch-like open (about 8 cm of separation between the two fingers), but not stretched.

Twenty MEPs have been collected on each posture, and the order of tested posture was counterbalanced across participants.

#### EMG Recording

We collected MEPs, by measuring EMG response to a single TMS pulse delivered to the M1 cortex by a BiStim^2^ stimulator (The Magstim Co. Ltd) equipped with a D70 Alpha coil (The Magstim Co. Ltd). EMG was recorded from the FDI and APB of the right hand to monitor hand intrinsic muscles primarily controlling the index finger and the thumb movements, by using B10-S-100 Ag/AgCl sintered ring electrode with 1, 5 mm touch proof safety socket, D360 amplifier (Digitimer, Hertfordshire, UK), Power1401 A/D converter, and Signal 5.08 software (Cambridge Electronic Design Limited, Cambridge, UK).

Resting motor threshold (rMT) was determined as the minimum single-pulse intensity required to produce at least 5 out of 10 MEPs greater than 50 μV in the left FDI, while participants kept their arm on the armrest in a resting position.

MEPs were obtained by stimulating the participants in the spot individuated with rMT procedure with the coil with a posteroanterior orientation, setting the intensity to 120% to the rMT.

EMG responses were processed following this procedure:

1. The RMS of activity in the time window of 1800 ms before the TMS pulse was taken as a measure of the muscular tonic activation. 50 ms pre-stimulus was not computed.
2. MEPs were calculated extracting the peak-to-peak response evoked by the TMS pulse in a time window of 10–40 ms post-stimulus. MEPs that did not evoke an amplitude of at least 0.2 mV were rejected, during the conditions where the posture was actively maintained. Rejection rate for Experiment 2 was 0%.

To demonstrate higher cortical excitability in one hand posture independent of the pre-stimulus tonic muscle activity needed to hold that posture, we had to compare the posture-related differences in the basal and TMS-induced EMG activity. Since these EMG activities are on different scale, we had to apply a normalization.

1. RMS and MEP responses were then standardized by dividing the responses in one posture by the responses in the other posture. Specifically, we divided the thumb-down by the thumb-up posture.

By doing so, MEPs and RMSs become directly comparable to being expressed as a proportion of change in respect to the posture held. Values were then log-transformed to avoid asymmetric distribution typical of ratios. Values bigger than 0 indicate thumb-down posture facilitation, while values smaller than 0 suggests facilitation induced by the thumb-up posture.

#### Analysis

Inferential statistics were performed through LMMs with the statistical software R (Team RC 2013), using the package lme4. Standardized EMG responses were analyzed as dependent variable. Participants were added to the model as random effect variable. The model was analyzed with an ANOVA with Satterthwaite approximation for degrees of freedom.

We entered, as fixed-effect model, a 2X (TMS: MEP/RMS) 2X (Muscle: FDI/APB) full factorial design.

To investigate significant effects, we calculated the 95% confidence intervals (CIs) of the diverse conditions. The facilitation for a specific posture is supported by the data if the CI does not include the value 0.

### Experiment 3

Experiment 2 was controlled for pre-stimulus muscle activity. However, in the case that other peripheral factors—linked to the two different assumed biomechanics—would have influenced Experiment 2 results, we designed Experiment 3 to surpass this limit. Indeed, in all the condition of Experiment 3, the task was only performed mentally, while the hand, the forearm, and the arm were at rest, in the very same posture in every condition.

Participants were asked to keep their right arm resting on the armrest in a comfortable relaxed position with the hand in a resting neutral position with the palm down and all the fingers relaxed and aligned on the horizontal plane of the armrest.

**Figure 3:**
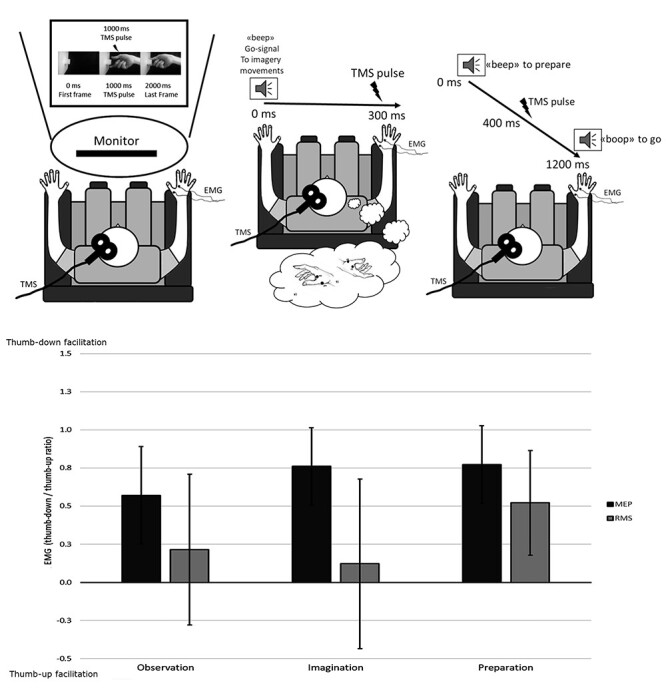
Experiment 3 setup and results. The upper panels show the pre-motor task conditions. Observation (left): The action was observed in short video clips of pinch grips on a monitor. Imagination (middle): The thumb-down/-up pinches had to be imagined. Preparation (right): The action was prepared at first and released only after a go-signal. The lower panel shows the results. Bars represent the average standardized EMG activity (EMG thumb-down/EMG thumb-up). Error lines indicate 95% CIs. When the CIs are above the *x*-axis, there is evidence that thumb-down posture is facilitated (e.g., MEPs in all the three tasks). When the CIs cross the axis, there is no facilitation for one of the two postures.

Participants were involved in three premotor representation of action tasks, during which MEPs were collected: 1) observation, 2) imagination, or 3) preparation. All these three conditions featured the same two hand postures of Experiment 2, and MEPs were collected following the Experiment 2 procedure except that in this case the hand was in a resting position so that MEPs that did not evoke an amplitude of at least 0.05 mV were rejected as non-evoked responses (Fig. 3). Rejection rate for Experiment 3 was 5.16% for observation, 3.05% for imagination, and 4.38%for preparation.

The order of the conditions was randomized across the participants, as well as the order of the tested postures. For each task and each posture, the 20 trials were performed consecutively.

The action to observe, imagine, or prepare was a pinch grip toward a cube block that could have been done with the thumb-up or thumb-down posture. The height of the cube was adjusted in such a way that participants judged the action equally easy with both hands configurations.

#### Observation

For the observation task, we recorded two videos of a right hand performing a precision grip action toward a wooden cube. The hand in the video was the same for all the participants, and was the hand of one of the experimenters, so not the biological hand of any participant.

The cube side was of 4 cm, and it was attached to a wooden panel. By doing so, the action resulted completely isolated from any context or background. Participants only saw a right hand entering the scene from the right side, already oriented in the required posture, grasping the cube with one of the two postures. TMS pulse was delivered 1000 ms before the first touch of the hand with the block when the grip posture was very clear. Video clips were recorded in 720p definition with 30 frames/s with a camera (GoPro HERO4 Silver, GoPro, San Mateo, CA). Video clips were recorded and edited in such a way to last 2000 ms, and the grasping action was simultaneous in the clips with the two postures.

For each posture, 20 trials per condition were recorded.

#### Imagination

In the imagination condition, we asked participants to imagine the precision grip action toward the wooden block. A pure sound lasting 300 ms was used as go-signal. The instruction was to imagine the grip with one of the two postures and to start the imagery movement only after the sound. TMS pulse was synchronized with the go-signal delivering the stimulation 300 ms after the sound. The inter-trial interval was of 3500 ms.

We recorded 20 imagery actions asking to imagine the thumb-up and 20 the thumb-down posture in a block design, counterbalancing which posture has to be imagined first, across the participants.

#### Preparation

In the preparation condition, we asked participants to respond to two different pure sounds separated by 1200 ms. The first sound indicated to the participant to prepare the action, while the second sound indicated to release the action and perform it. The actions were the same of observation and imagination conditions. TMS pulse was given during the preparation phase 400 ms after the first sound.

For each posture, we recorded 20 trials per condition.

#### Analysis

The same statistical approach of *Experiment 1* and *2* was adopted, namely a LMM with the statistical software R (Team RC 2013) using the package lme4. The dependent variable was the standardized EMG responses. Participants were added to the model as random effect variable. The model was analyzed with an ANOVA with Satterthwaite approximation for degrees of freedom.

Here we entered, as fixed-effect model, a 2X (*TMS*: MEP/RMS) 2X (Muscle: FDI/APB) 3X (pre-motor task: observation/imagination/preparation) full factorial design.

Additionally, the three tasks were analyzed independently in a sequence of three independent 2X (TMS: MEP/RMS) 2X (Muscle: FDI/APB) factorial designs.

To explore the meaning of significant effects and interactions, we calculated the 95% CIs. The same interpretation of Experiment 2 is valid: Values bigger than 0 suggest facilitation for the thumb-down posture and values smaller than 0 indicate facilitation for the thumb-up posture.

### Experiment 4

The SAI TMS protocol, likely measuring cholinergic cortical inhibition (Di Lazzaro et al*.* 2000), was employed to assess whether the corticospinal facilitation of one posture could be due to the different sensory afferent input, in line with a sensory–motor interaction hypothesis. SAI responses were collected in the same two hand postures adopted in Experiment 2: thumb-down and thumb-up. The first posture to hold was counterbalanced across the participants. Each SAI procedure included 50 MEP recordings (see below).

At first, the precise timing of individual N20 component elicited by the stimulation of the median nerve was identified. The N20 component is considered the first cortical component evoked by somatosensory stimulation, and it is located in primary somatosensory cortex (S1). To measure the N20 latency, we adopted an electrical stimulator (Digitimer DS7A, Hertfordshire, UK) delivering stimuli to the median nerve of the right arm at the wrist level at a frequency of 4.9 Hz.

The recording electrode was placed on S1 area, reference one on earlobe ipsilateral to the electrical stimulation, and ground one on dorsal part of wrist (B10-S-100 Ag/AgCl sintered ring electrode). N20 was averaged following 500 stimulations, resulting in a procedure lasting less than 3 min. The electroencephalographic signals were recorded by using D360 amplifier (Digitimer, Hertfordshire, UK), Power1401 A/D converter (CED, Cambridge Electronic Design Limited, Cambridge, UK), and Signal 5.08 software (Cambridge Electronic Design Limited, Cambridge, UK).

TMS intensity and hotspot were determined as in Experiment 2; except that in this case, we set the intensity at the 110% of the rMT.

The SAI follows a fixed procedure where standard MEPs are mixed with conditioned MEPs. The protocol that we followed was composed of 50 TMS stimulations. The first 10 trials were collected without a concurrent median nerve electrical stimulation as well as the last 10 (i.e., unconditioned MEP trials). The remaining 30 trials are conditioned by concurrent electrical stimulation of the median nerve. The median nerve stimulation was synchronized with the TMS pulse in such a way that the TMS followed the median nerve stimulation by the latency of the individual N20 + 2 ms (10 trials), +3 ms (10 trials), or +4 ms (10 trials).

The effect of the SAI is calculated subtracting 1 to the ratio of the MEPs evoked during the conditioned trials by the unconditioned trials and multiplied by 100. By doing so, the decrease of the amplitude is expressed in percentage of the unconditioned trials (Di Lazzaro et al. 2007; Fischer and Orth 2011). For example, a value of −30% indicates that the conditioned response is 30% less of the unconditioned response. Following this procedure, values smaller than 0 indicate that the median nerve stimulation reduced the amplitude of the MEPs (i.e., the SAI procedure was effective); thus, it has inhibited the corticospinal excitability.

For each participant, the SAI procedures were run twice: first while holding the thumb-up posture and second with the thumb-down posture.

#### Analysis

Trials that did not evoke MEPs with an amplitude of at least 0.2 mV (0.05%) were rejected.

Since the outcome of SAI is a single data point per participant, LMM could not be used as in Experiments 1–3. Therefore, we adopted a more classic repeated measure ANOVA design, calculated with the software JASP 0.9.1 (JASP Team 2018).

The percentage of decreased response, calculated as described above, was the dependent variable.

We tested a 2X (Hand posture: Thumb-Down/Thumb-Up) 2X (Muscle: FDI/APB) full factorial design.

Once again, significant effects have been explored by using the 95% CIs.

## Results

### Behavioral Advantage of Hand Posture in Action

In Experiment 1, we measured the onset of a pinch action of a wooden cube performed with the thumb up, or down; we also measured the time to actually perform the entire action. We found that the pinch started earlier when it was done with the thumb-down configuration showing a behavioral advantage in the computation of the action plan (main effect of factor hand posture (*F*(1,5498.1) = 127.460, *P* < 0.001; interaction hand posture ^*^ forearm orientation (*F*(1,5498.1) = 15.694, *P* < 0.001)). The fastening effect for the thumb-down pinch was stronger if the participants had to start the pinch with a prone orientation of the forearm (prone forearm CIs: thumb-down = 292 ms, 342 ms; thumb-up = 319 ms, 367 ms). Nonetheless, the temporal advantage was detectable also when starting the action with a reversed orientation of the arm (supine forearm hand posture CIs: thumb-down = 324 ms, 372 ms; thumb-up = 337 ms, 385 ms), suggesting that the computational advantage for the hand posture is not determined by the synergic advantage of the position of the whole arm (Fig. 1).

Moreover, the advantage of the thumb-down pinch was not limited to the initial computation of the action program. We isolated the reaching component subtracting the time to initiate the action from the time to pinch the wooden cube. The reaching component of the action was faster when it was done with the thumb-down posture (main effect of factor *hand posture* (*F*(1,1839.01) = 64.062, *P* < 0.001), no matter the starting orientation of the forearm (Prone forearm CIs: thumb-down = 502 ms, 626 ms; thumb-up = 528 ms, 652 ms. Supine forearm CIs: thumb-down = 501, 627 ms; thumb-up = 538 ms, 662 ms) (Fig. 1).

### Increased Corticospinal Excitability Holding Hand Postures

To make evoked and basal muscle activity comparable, they were computed as ratio between their values recorded for the thumb-down by the thumb-up posture (EMG_thumb-down_/EMG_thumb-up_). The ratio is then log-transformed to linearize the values. The log-transformed ratio was used as response variable. Values bigger than 0 indicate a facilitation for the thumb-down posture and values smaller than 0 indicate a facilitation for the thumb-up posture. Moreover, to better account for participant variability, the ANOVA was performed on LMM, modeled with participants as random effect variable and the studied muscle (factor Muscle: FDI vs. APB) and the evoked or basal muscle activity (factor *TMS*: MEP vs. RMS) as predictors.

In Experiment 2, MEPs changed significantly in the two postures, depending on the studied muscle, while RMS did not (TMS × Muscle interaction *F*(1,1261) = 8.177, *P* < 0.01). The facilitation for a specific posture appeared only in the FDI muscle for the MEP as showed by the inspection of 95% CI that did not include the value 0 only for the FDI muscle (CIs: FDI = 0.519, 1.272; ABP = −0.044, 1.007). Crucially, basal muscle activity (RMS) was not affected by posture neither for FDI (CI = −0.243, 0.927) nor for APB (CI = −0.874, 0.989), revealing that the facilitation of the corticospinal excitability was independent of basal tonic activity (Fig. 2). We also observed a main effect of the factor TMS (*F*(1,1261) = 9.857, *P* < 0.01) that is better understood looking at the abovementioned interaction.

### Increased Corticospinal Excitability Mentally Representing Hand Postures

In Experiment 3, we added a third predictor with three levels specifying for the task condition: observation, imagination, and preparation of the action (main factor: pre-motor *task*). Notably, in Experiment 3, the actual posture of the hand was at rest in a neutral position: relaxed on the armrest, open, with the fingers aligned on the vertical plane (i.e., no relative up or down position for one finger than the others).

The MEPs changed significantly in the two postures, while the RMS did not (main effect of factor TMS: *F*(1,3658.9) = 23.021, *P* < 0.001). Crucially, the MEPs were larger in the thumb-down pinch (CI = 0.428, 0.978), while the RMSs were not (CI = −0.144, 0.746), suggesting that also when the thumb-down posture is just mentally represented, it induces the facilitation of the corticospinal excitability independently from basal tonic activity (Fig. 3).

This effect did not interact with the factor Muscle, and more importantly, it did not interact with the factor pre-motor task (all *P* values >0.23), suggesting that the effect found is independent also from the level of motor representation.

Additionally, the main effect of pre-motor task was significant (*F*(1,3658.9) = 3.130, *P* = 0.045), showing that EMG is larger during motor preparation condition (0.362, 0.945) than the other two: motor imagery (0.142, 0.839) and motor observation (0.021, 0.795). However, this effect did not interact with the hand posture.

Indeed, the facilitation for the thumb-down posture was confirmed by the results of the independent analysis of the three pre-motor tasks. In all the three tasks, we found that MEPs were larger than 0 (MEPs CI: observation = 0.252, 0.889; imagination = 0.506, 1.015; preparation = 0.518, 1.026), supporting the facilitation for the actions mentally represented with the thumb-down (Fig. 3). In agreement with Experiment 2, the MEPs were significantly different than RMS (main effect of factor TMS— observation: *F*(1,1195.1) = 6.557, *P* = 0.01; imagination: *F*(1,1201.7) = 8.118, *P* < 0.01; preparation: *F*(1,1213.2) = 23.352, *P* < 0.001), strengthening the idea that the motor facilitation is independent from the pre-TMS tonic activation.

Moreover, only in the preparation task, we found that also RMS had an advantage for the thumb-down posture, as the CI (0.179, 0.864) did not include 0. However, the postural advantage does not seem to be dependent from the task (no interaction task ^*^ TMS *F*(2,3658.9) = 1.45, *P* = 0.23), and the advantage seen for RMS thumb-down posture in preparation may be result of an additive, noninteractive, effect of the task plus the posture.

### Somatosensory Modulation of Motor Activity Is Not Posture-Dependent

In Experiment 4, the effect of SAI is expressed as the percentage change of conditioned average MEP compared to average unconditioned MEP; negative values mean that conditioned MEP is inhibited. As expected, SAI was effective in modulating the motor cortex excitability in all postures and muscles studied (CI: thumb-up/FDI = −46.45%, −21.0%; thumb-down/FDI = −40.5%, −23.7%; thumb-up/APB = −36.7%, −10.3%; thumb-down/APB = −35.3%, −13.3%). APB was slightly less inhibited (−36.0%, −11.8%) than FDI (−43.4%, −22.4%) (significant main effect, Muscle: *F*(1,13) = 4.911, *P* = 0.045), probably because TMS was delivered on the FDI hotspot and it has been demonstrated that SAI is maximal at the cortical hotspot of the tested muscle (Dubbioso et al. 2017).

**Figure 4:**
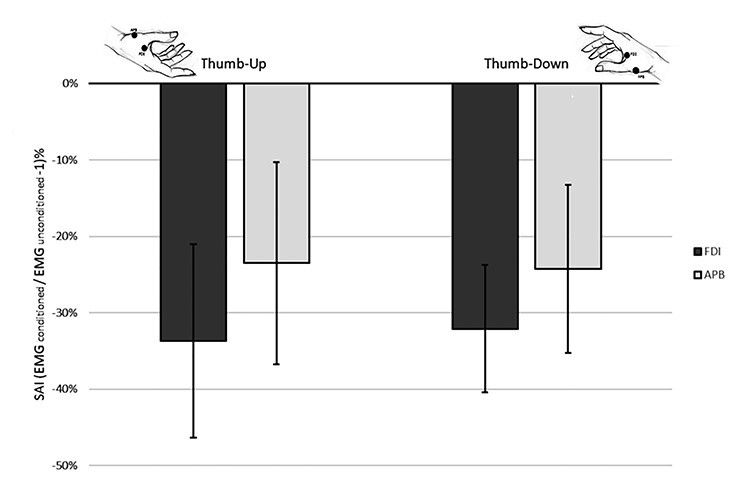
Experiment 4 results. Bars represent the average SAI effect calculated as a percentage of change of the conditioned trials to the unconditioned trials (SAI = (MEP_conditioned_/MEP_unconditioned_ − 1) ^*^ 100). Error lines indicate the 95% CIs. The afferent median nerve stimulation inhibited motor cortex excitability, but this was independent from the hand posture (i.e., thumb-up and thumb-down inhibition is comparable).

Crucially, the effect of SAI was not different in the two postures (*F*(1,13) = 0.005, *P* = 0.945), neither postures did interact with the muscle studied (*F*(1,13) = 0.101, *P* = 0.756) (Fig. 4). This suggests that the facilitation induced by the thumb-down posture, found in Experiments 2 and 3, is independent of contingent sensory afferences.

## Discussion

In this study, we investigated the hypothesis of the existence of a hand posture favored by central processes impacting action planning and execution.

It is known that body posture may affect the spatial processing of sensory stimuli, because a computational effort is needed to re-reference multiple streams of afferent and/or efferent information in a common frame, based on the internal representation of the body. For example, in the classic hand laterality task, when participants discriminate if a displayed hand is left or right, RTs increase if participants’ hand posture and the displayed hand do not match (Parsons 1987). On the contrary, when body spatial information coming from proprioception and vision match, a transformation is spared and the computational effort is reduced, thus the spatial processing of sensory stimuli is known to be favored and, in turn, it facilitates motor imagery (Parsons 1987; Azañón and Soto-Faraco 2008). In this imagined motor control task, the frontoparietal network increases its activity accordingly with complexity of the targeted movement and with the difference from the actual hand posture (de Lange et al. 2006).

Along the same line, we hypothesized that similar facilitation could be obtained also by aligning the sensory–motor information coming from the hand with a given particular way to represent the hand in the brain. We verified this hypothesis looking for a posture-related behavioral advantage performing an action and for an increased motor cortex excitability when the hand is mentally represented or actually held.

We found strong and consistent facilitation of the motor system for the pinch grip posture with the thumb-down, compared with its opposite. The facilitation was not only strong, but also it spread to different domains, tested with various experiments, which all gave concordant results in favor of the thumb-down pinch.

### Behavioral Advantage of Thumb-Down Pinch Posture

Experiment 1 demonstrated a behavioral advantage of the thumb-down pinch in two aspects. 1) Neural computation was faster when the thumb-down pinch grip was performed, as shown by the shorter interval from the cue signal to the onset of muscle activation. This advantage was independent of the phase of the grip as measured through sensors placed in the hand, forearm, and arm, monitoring the reaching, shaping, and grasping phases. 2) The thumb-down pinch grip was also faster to reach the target, independently from the shorter movement onset. Moreover, the thumb-down shorter times were detectable either starting from a prone forearm posture, or a supine forearm posture, despite the starting position had a facilitation role in the time to onset and not on the reaching time.

To note, Experiment 1 results, especially the shorter time to onset, cannot be due to biomechanical or other peripheral factors.

In this task, three postures have to be considered: 1) the one assumed by the internal representation of the hand (thumb-up/thumb-down), 2) the actual starting forearm orientation when the movement is planned, and 3) the posture targeted by the pinch grasping.

When an action congruent with the favorite internal representation (thumb-down) was asked starting from the pronated forearm, no transformation was required to align the different postures because that action was congruent with both the internal and the actual posture. This resulted in the most efficient processing at the onset time.

Instead, when participants were asked to perform a thumb-up pinch starting from a pronated forearm, they had to resolve two incongruencies: from the internal favorite representation and from the actual position. The slower RT would reflect the processing time needed to align the different postures.

Differently, starting from a supinated forearm posture, always introduces one discrepancy: from the internal representation when the pinch required has the thumb-up and from the actual posture when the pinch required has the thumb-down.

The presence of the hand posture ^*^ forearm orientation interaction only for the time to onset highlights how the advantage of matching postures is especially manifested in the motor programming phase.

### Motor Cortex Facilitation of Thumb-Down Pinch Posture

Experiment 2 showed that, while holding the thumb-down pinch posture, the excitability of the primary motor cortex was enhanced. Comprehensively, the congruency between behavioral advantage and the one showed by enhanced corticospinal excitability gives strength to our results. MEP enhancement was not due to different ongoing muscular and spinal loop activity, because its increase was significantly higher than any increase seen in the tonic activity of the muscle recorded before the TMS stimulus. However, to have a further proof of the cortical origin of our findings, and of their independence from actual hand–arm posture-related factors (e.g., extent of wrist stretch), we run Experiment 3.

Specifically, Experiment 3 showed the same motor facilitation for the thumb-down posture when the hand was kept stationary in a resting neutral position, no actual movement was required, and hand posture was only representational. Interestingly, motor facilitation was found when the hand action was either observed, imagined, or planned. The facilitation was found both when those three levels were modeled altogether and when each of them was modeled independently.

Noteworthy, when the posture was actually held stationary, as in Experiment 2, the advantage was significant only for the FDI muscle. Conversely, when the entire action involving also the shaping phase of the pinch was mentally represented, as in Experiment 3, the advantage for the thumb-down configuration became significant for both APB and FDI. This well fits with the involvement of APB only in the shaping phase, while it is not required by holding the posture steadily.

The equivalence theory suggests that the motor processing of action observation and motor imaging are equivalent with the one of actual physical execution, while an inhibitory process blocks the motor command before it is sent downward to the muscles (Jeannerod 2001; Guillot et al. 2012). Accordingly, by investigating M1 excitability in those conditions, we tested the neural processes in charge of motor processing unchained by the constraints of physical execution. Results of Experiment 3 suggest that the effect of motor facilitation when the hand pinches with the thumb-down and all other fingers up relies on central mechanisms, possibly related to deeply rooted motor representation quite early on, before the level of motor output.

### A Common Standard Posture of the Hand for Sensory and Motor Processing

A similar postural advantage for information processing was previously found for the somatosensory system (Romano et al*.* 2017, 2019; Manser-Smith et al. 2020).

Two hypotheses could explain the relationship between sensory and motor systems. On one side, the motor advantage could be a balancing of concurrent afferent inhibition because the activation of the sensory system is known to inhibit the motor output, as in the SAI protocol (Tokimura et al. 2000), and vice versa motor system activation reduces somatosensory cortical activity, as in the motor gating of SEP (Rushton et al. 1981; Seki and Fetz 2012). In this case, the postural advantage for the motor system should be for the opposite configuration than the one found for sensory tasks. However, the pinch posture we found facilitated for action was the same that we previously found facilitated for the processing of somatosensory stimuli: Tactile stimuli delivered to the thumb were discriminated faster and more accurately when it is held in a relatively low elevation, while stimuli delivered to any other finger were better discriminated when the finger occupies a relatively high position (Romano et al. 2017). Moreover, in Experiment 4, the SAI did not highlight a posture when motor cortex excitability was preferentially inhibited, making unlikely the possibility that motor facilitation is due to lower sensory inhibition.

On the other hand, the consistency between the favored postures in sensory and motor systems and the absence of a different sensory-driven inhibition in the thumb-up and opposite posture support an alternative, and more intriguing, hypothesis: The hand posture-related advance we found is independent, likely hierarchically above, to any specific sensory modality or motor processing.

Overall, the present findings are strongly in favor of the origin of the reported sensorimotor facilitation in higher-order frontal areas or integrative parietal regions, namely those that come into play in the motor control network (Miall and Wolpert 1996; Flanagan and Wing 1997; Naito et al. 2016) and in the body representation (Berlucchi and Aglioti 1997; Rushworth et al. 2003; Ehrsson et al. 2004; Berlucchi and Aglioti 2010).

Enhanced corticospinal excitability and decreased RT have been described as a consequence of decreasing the uncertainty of a required action and have been explained in a Bayesian framework as the strengthening of the prior to that action (Bestmann et al. 2008).

## General Discussion

Why, in our case, a particular hand posture would induce such a strong and consistent sensorimotor processing facilitation, independent from the actual biomechanics, sensory feedback, or other peripheral factors? Should we also have a prior facilitating that posture? We speculate that such posture may serves as, or it is close to, the a priori representation of the hand in the brain.

Indeed, the body representation could be continuously built by sensory feedback, as an image in a TV screen is built on the information flow and ceases to exist when the stream is interrupted. Alternatively, body representation could exist as an a priori internal representation and be only, but still continuously, modulated by sensory afferent input. In this latter case, there should be an a priori posture in which the body is represented. Matching the posture a priori represented in the brain to the actual posture of the body or to the one targeted by a movement would result, respectively, in faster sensory and motor processing.

Indeed, our results well fit with the idea that the brain contains a baseline spatial configuration of the representation of the body; a standard posture that works as a Bayesian prior, in other words, as a baseline reference functional to guide any subsequent body-space-related perception and action.

The possibility that the brain represents the body with a “standard” posture has fascinated scientists for a long time (Longo 2015). In the 1970s, Melzack and Bromage found that after deafferentation of the brachial plexus or subarachnoid anesthesia participants felt “phantom” body parts holding few stereotyped postures (Melzack and Bromage 1973; Bromage and Melzack 1974). However, those seminal results have been challenged when other authors failed to replicate their findings (Inui et al. 2011); the limited evidence left the question unsolved and the hypothesis relatively abandoned. Research on body representation has mostly focused on its flexible nature. For example, the striking Rubber Hand Illusion, which helped so much in understanding the dynamic processes of body representation, clarified how visuotactile and proprioceptive feedback integration constantly updates body representation in order to localize and recognize one’s own body in space (Botvinick and Cohen 1998; Frith et al. 2000; Tsakiris 2010). However, studies on body representation dynamic modifications neither provide clues on the possibility of the existence of an a priori representation of the body nor help to depict how the body is postured in this a priori representation.

Considering modeling approaches to motor control, the body representation is seen as an internal model, which is exploited to predict the variation of the state of the body at the next time point given the input at the current time; this prediction is fused with the sensory feedback to provide the best estimation of body state using the prediction error (i.e., the difference between predicted body state and the one measured by the sensory feedback) as correction signal.

How such process would be facilitated when the desired target posture match, or is close to, an a priori representational posture?

In this view, internal model should include the computation of a high-dimensional dynamic model accounting for all parts of the body in all possible configurations, which would require a massive computational effort. Thus, several hypotheses, such as the motor primitive one (Hogan and Sternad 2012), have been done to reduce the dimensionality of the computation. The proximity between target posture and represented posture could be another way to reduce such dimensionality, because the error between the initial guess, given by the a priori posture, and the sensory feedback would be quicker to converge to zero.

Moreover, multisensory integration and computing an inverse model of the body are needed already at the motor planning stage (Sober and Sabes 2003); thus, it may be hypothesized that planning to perform a grip that matches with the posture in which the hand is represented would require at this stage lower computational costs. This may happen because any further transformation from the internal to the target posture would not be necessary, or as if, given the internal posture, the targeted posture has lower uncertainty (Bestmann et al*.* 2008). Thus, in line with our findings, the motor system would be more prone and faster to perform a grasping action requiring a posture closer to the represented one.

An important aspect of our study is that the preferred posture was not determined by action goals or object affordance, which may facilitate a given hand configuration for grasping (Jeannerod 1988; Sartori et al. 2011), given that the tasks used were neutral to these aspects. Indeed, there is no clear evidence that one posture is more frequently used than the other because specific actions may require one or the other; for example, texting on a smartphone requires typically the thumb above the other fingers, while pinching a pen aligns the thumb with the index finger, and eating a sandwich requires the thumb below the other fingers.

In the absence of an environmental trigger, between hand configurations equally easy to adopt, tested participants still hold a preferred grip posture that facilitates the motor system, also when the hand was held at rest in a neutral position and action was only imagined.

We tested the same pinch in two opposite postures, one with the forearm pronated and the other with the forearm supinated, to maximize the possibility that one of the two was advantaged because closer to how the hand is a priori represented in the brain. Clearly, we did not test every possible hand posture; thus, it is possible that the orientation of the hand a priori representation may not be exactly the one that we tested. Nevertheless, we can affirm that all the postures are not equivalent and that the preferred one is closer to the thumb-down grip posture than the thumb-up one.

Why the a priori hand representation has this specific configuration? Is the standard posture innate, or learned? These are crucial and still open questions.

At present, since the posture that we found to be facilitated is not associable with a prototypical grasping for all everyday actions, its sensorimotor advantages in the interaction with the environment may have an evolutionary explanation.

In the full-hand power grip, the side of the pinch where the second to fifth fingers are placed is stronger than the side where the thumb is; this would be functional to fight against a force directed down-up. In evolutionary terms, what we call standard posture may have given advantages to primates bouncing from branch to branch during arboreal locomotion (Fang et al. 2014). On the contrary, when humans started to live on the ground, most of the forces were directed up-down, as when lifting objects. In the standard posture, only the thumb antagonizes up-down forces, a disadvantage in activities requiring great force. However, being the second to fifth fingers not busy to fight against loads, they were free to perform fine and dexterous movements, from sewing to piano playing. Thus, the sensorimotor advantage of standard posture may have contributed to the development of human manipulation, a crucial functional advantage for human evolution.

## Conclusion

We started the present study by questioning the existence of hand posture favored in its central motor processing. We can conclude that all the postures are not equivalent, and thus it is likely that there is a favorite one. Our results showed that the thumb-down grip, compared with its opposite (thumb-up) posture, favors central computation, increases motor cortex excitability, and gives behavioral advantages. This posture is the same that facilitates tactile discrimination.

We hypothesized that the origin of this advantages likely resides in the match between the facilitated posture and the one held by the internal representation of the hand. These findings overall suggest the existence of a representation of hand posture for perception and action that might be a baseline reference for any body–space interaction, working as a Bayesian prior in biasing subsequent processing.

The potential discovery of this prior may have important applicative impact. For instance, it may help the understanding of pathological motor control of the hand, for example, following stroke, and guide its rehabilitation (Di Pino, Pellegrino, et al. 2014b). It may also inform the development of dexterous hand prostheses, where the implementation of a low-level internal control loop that complies with standard posture facilitation (Di Pino, Maravita, et al. 2014a; Zollo et al. 2019; Di Pino et al. 2020) would result in more functional and possibly embodiable prostheses.

## Data Availability

The datasets generated during this study are available at Mendeley Data (Dataset_Romano_CerebralCortex_2020).

## References

[ref1] Azañón E , Soto-Faraco S. 2008. Changing reference frames during the encoding of tactile events. Curr Biol. 18:1044–1049. 18619841 10.1016/j.cub.2008.06.045

[ref2] Berlucchi G , Aglioti S. 1997. The body in the brain: neural bases of corporeal awareness. Trends Neurosci. 20:560–564. 9416668 10.1016/s0166-2236(97)01136-3

[ref3] Berlucchi G , Aglioti SM. 2010. The body in the brain revisited. Exp Brain Res. 200:25. 19690846 10.1007/s00221-009-1970-7

[ref4] Bestmann S , Harrison LM, Blankenburg F, Mars RB, Haggard P, Friston KJ, Rothwell JC. 2008. Influence of uncertainty and surprise on human corticospinal excitability during preparation for action. Curr Biol. 18:775–780. 18485711 10.1016/j.cub.2008.04.051 PMC2387198

[ref5] Botvinick M , Cohen J. 1998. Rubber hands ‘feel’ touch that eyes see. Nature. 391:756–756. 9486643 10.1038/35784

[ref6] Bromage PR , Melzack R. 1974. Phantom limbs and the body schema. Can Anaesth Soc J. 21:267–274. 4838325 10.1007/BF03005731

[ref8] de Lange FP , Helmich RC, Toni I. 2006. Posture influences motor imagery: an fMRI study. NeuroImage. 33:609–617. 16959501 10.1016/j.neuroimage.2006.07.017

[ref9] De Vignemont F . 2010. Body schema and body image—pros and cons. Neuropsychologia. 48:669–680. 19786038 10.1016/j.neuropsychologia.2009.09.022

[ref10] Di Lazzaro V , Oliviero A, Profice P, Pennisi M, Di Giovanni S, Zito G, Tonali P, Rothwell J. 2000. Muscarinic receptor blockade has differential effects on the excitability of intracortical circuits in the human motor cortex. Exp Brain Res. 135:455–461. 11156309 10.1007/s002210000543

[ref11] Di Lazzaro V , Pilato F, Dileone M, Profice P, Ranieri F, Ricci V, Bria P, Tonali P, Ziemann U. 2007. Segregating two inhibitory circuits in human motor cortex at the level of GABAA receptor subtypes: a TMS study. Clin Neurophysiol. 118:2207–2214. 17709293 10.1016/j.clinph.2007.07.005

[ref12] Di Pino G , Maravita A, Zollo L, Guglielmelli E, Di Lazzaro V. 2014a. Augmentation-related brain plasticity. Front Syst Neurosci. 8:109. 24966816 10.3389/fnsys.2014.00109 PMC4052974

[ref13] Di Pino G , Pellegrino G, Assenza G, Capone F, Ferreri F, Formica D, Ranieri F, Tombini M, Ziemann U, Rothwell JC, et al. 2014b. Modulation of brain plasticity in stroke: a novel model for neurorehabilitation. Nat Rev Neurol. 10:597–608. 25201238 10.1038/nrneurol.2014.162

[ref14] Di Pino G , Romano D, Spaccasassi C, Mioli A, D’Alonzo M, Sacchetti R, Guglielmelli E, Zollo L, Di Lazzaro V, Denaro V, et al. 2020. Sensory- and action-oriented embodiment of neurally-interfaced robotic hand prostheses. Front Neurosci. 14:1–17. 10.3389/fnins.2020.00389 PMC7232597 32477046

[ref15] Dubbioso R , Raffin E, Karabanov A, Thielscher A, Siebner HR. 2017. Centre-surround organization of fast sensorimotor integration in human motor hand area. NeuroImage. 158:37–47. 28669907 10.1016/j.neuroimage.2017.06.063

[ref16] Ehrsson HH , Spence C, Passingham RE. 2004. That's my hand! Activity in premotor cortex reflects feeling of ownership of a limb. Science (New York, NY). 305:875–877. 10.1126/science.1097011 15232072

[ref7] Fang C, Jiang T, Yuan X. 2014. Human bipedalism, evolved from arboreal locomotion of two-arm brachiation.

[ref17] Fischer M , Orth M. 2011. Short-latency sensory afferent inhibition: conditioning stimulus intensity, recording site, and effects of 1 Hz repetitive TMS. Brain Stimul. 4:202–209. 22032735 10.1016/j.brs.2010.10.005

[ref18] Flanagan JR , Wing AM. 1997. The role of internal models in motion planning and control: evidence from grip force adjustments during movements of hand-held loads. J Neurosci. 17:1519–1528. 9006993 10.1523/JNEUROSCI.17-04-01519.1997 PMC6793733

[ref19] Frith CD , Blakemore S-J, Wolpert DM. 2000. Abnormalities in the awareness and control of action. Philos Trans R Soc Lond Ser B Biol Sci. 355:1771–1788. 11205340 10.1098/rstb.2000.0734 PMC1692910

[ref20] Guillot A , Di Rienzo F, MacIntyre T, Moran A, Collet C. 2012. Imagining is not doing but involves specific motor commands: a review of experimental data related to motor inhibition. Front Hum Neurosci. 6:247. 22973214 10.3389/fnhum.2012.00247 PMC3433680

[ref21] Hogan N , Sternad D. 2012. Dynamic primitives of motor behavior. Biol Cybern. 106:727–739. 23124919 10.1007/s00422-012-0527-1 PMC3735361

[ref22] Inui N , Walsh L, Taylor J, Gandevia S. 2011. Dynamic changes in the perceived posture of the hand during ischaemic anaesthesia of the arm. J Physiol. 589:5775–5784. 21946853 10.1113/jphysiol.2011.219949 PMC3249049

[ref23] Jeannerod M . 1988. The neural and behavioural organization of goal-directed movements. Oxford, UK: Clarendon Press/Oxford University Press.

[ref24] Jeannerod M . 2001. Neural simulation of action: a unifying mechanism for motor cognition. NeuroImage. 14:S103–S109. 11373140 10.1006/nimg.2001.0832

[ref25] Kawato M . 1999. Internal models for motor control and trajectory planning. Curr Opin Neurobiol. 9:718–727. 10607637 10.1016/s0959-4388(99)00028-8

[ref26] Longo MR . 2015. Implicit and explicit body representations. Eur Psychol. 20:6–15.

[ref27] MacKenzie CL , Iberall T. 1994. The grasping hand. In Advances In Psychology. Vol. 104. North Holland: Hardcover.

[ref28] Manser-Smith K , Romano D, Tamè L, Longo MR. 2021. Fingers hold spatial information that toes do not. Q J Exp Psychol. 74(1):95–105. 10.1177/1747021820960094 33016220

[ref29] Melzack R , Bromage P. 1973. Experimental phantom limbs. Exp Neurol. 39:261–269. 4702820 10.1016/0014-4886(73)90228-8

[ref30] Miall RC , Wolpert DM. 1996. Forward models for physiological motor control. Neural Netw. 9:1265–1279. 12662535 10.1016/s0893-6080(96)00035-4

[ref31] Naito E , Morita T, Amemiya K. 2016. Body representations in the human brain revealed by kinesthetic illusions and their essential contributions to motor control and corporeal awareness. Neurosci Res. 104:16–30. 26562333 10.1016/j.neures.2015.10.013

[ref32] Napier J . 1962. The evolution of the hand. Sci Am. 207:56–65. 13937573 10.1038/scientificamerican1262-56

[ref33] Parsons LM . 1987. Imagined spatial transformations of one's hands and feet. Cogn Psychol. 19:178–241. 3581757 10.1016/0010-0285(87)90011-9

[ref34] Romano D , Marini F, Maravita A. 2017. Standard body-space relationships: fingers hold spatial information. Cognition. 165:105–112. 28531806 10.1016/j.cognition.2017.05.014

[ref35] Romano D , Tamè L, Amoruso E, Azañón E, Maravita A, Longo MR. 2019. The standard posture of the hand. J Exp Psychol Hum Percept Perform. 45:1164–1173. 31144857 10.1037/xhp0000662

[ref36] Rushton DN , Rothwell JC, Craggs MD. 1981. Gating of somatosensory evoked potentials during different kinds of movement in man. Brain. 104:465–491. 7272711 10.1093/brain/104.3.465

[ref37] Rushworth M , Johansen-Berg H, Göbel SM, Devlin J. 2003. The left parietal and premotor cortices: motor attention and selection. NeuroImage. 20:S89–S100. 14597301 10.1016/j.neuroimage.2003.09.011

[ref38] Sartori L , Straulino E, Castiello U. 2011. How objects are grasped: the interplay between affordances and end-goals. PLoS One. 6:1–10. 10.1371/journal.pone.0025203 PMC3182194 21980396

[ref39] Seki K , Fetz EE. 2012. Gating of sensory input at spinal and cortical levels during preparation and execution of voluntary movement. J Neurosci. 32:890–902. 22262887 10.1523/JNEUROSCI.4958-11.2012 PMC3293372

[ref40] Sober SJ , Sabes PN. 2003. Multisensory integration during motor planning. J Neurosci. 23:6982–6992. 12904459 10.1523/JNEUROSCI.23-18-06982.2003 PMC6740676

[ref41] R Core Team. 2013. R: A Language and Environment for Statistical Computing. R Foundation for Statistical Computing, Vienna.

[ref42] Tokimura H , Di Lazzaro V, Tokimura Y, Oliviero A, Profice P, Insola A, Mazzone P, Tonali P, Rothwell JC. 2000. Short latency inhibition of human hand motor cortex by somatosensory input from the hand. J Physiol. 523(Pt 2):503–513. 10699092 10.1111/j.1469-7793.2000.t01-1-00503.x PMC2269813

[ref43] Tsakiris M . 2010. My body in the brain: a neurocognitive model of body-ownership. Neuropsychologia. 48:703–712. 19819247 10.1016/j.neuropsychologia.2009.09.034

[ref44] Wentink E , Schut V, Prinsen E, Rietman JS, Veltink PH. 2014. Detection of the onset of gait initiation using kinematic sensors and EMG in transfemoral amputees. Gait Posture. 39:391–396. 24001871 10.1016/j.gaitpost.2013.08.008

[ref45] Wolpert DM , Ghahramani Z. 2000. Computational principles of movement neuroscience. Nat Neurosci. 3(Suppl):1212–1217. 11127840 10.1038/81497

[ref46] Zollo L , Di Pino G, Ciancio AL, Ranieri F, Cordella F, Gentile C, Noce E, Romeo RA, Dellacasa Bellingegni A, Vadalà G, et al. 2019. Restoring tactile sensations via neural interfaces for real-time force-and-slippage closed-loop control of bionic hands. Sci Robot. 4:eaau9924. 31620665 10.1126/scirobotics.aau9924 PMC6795534

